# Chondrocytes Contribute to Alphaviral Disease Pathogenesis as a Source of Virus Replication and Soluble Factor Production

**DOI:** 10.3390/v10020086

**Published:** 2018-02-15

**Authors:** Elisa X. Y. Lim, Aroon Supramaniam, Hayman Lui, Peta Coles, Wai Suet Lee, Xiang Liu, Penny A. Rudd, Lara J. Herrero

**Affiliations:** 1 Institute for Glycomics, Griffith University, Gold Coast Campus, Southport, QLD 4215, Australia; elisa.lim@griffithuni.edu.au (E.X.Y.L.); aroon.supramaniam@griffithuni.edu.au (A.S.); hayman.lui@griffithuni.edu.au (H.L.); peta.coles@griffithuni.edu.au (P.C.); waisuet.lee@griffithuni.edu.au (W.S.L.); xiang.liu@griffithuni.edu.au (X.L.); p.rudd@griffith.edu.au (P.A.R.); 2 School of Medicine, Griffith University, Gold Coast Campus, Southport, QLD 4215, Australia

**Keywords:** cartilage degradation, viral arthritis, inflammatory disease

## Abstract

Arthritogenic alphavirus infections often result in debilitating musculoskeletal disorders that affect the joints, muscle, and bone. In order to evaluate the infection profile of primary human skeletal muscle and chondrocyte cells to Ross River virus (RRV) in vitro, cells were infected at a multiplicity of infection (MOI) of 1 over a period of two days. Viral titers were determined by plaque assay and cytokine expression by Bio-Plex^®^ assays using the supernatants harvested. Gene expression studies were conducted using total RNA isolated from cells. Firstly, we show that RRV RNA is detected in chondrocytes from infected mice in vivo. Both human primary skeletal muscle and chondrocyte cells are able to support productive RRV infection in vitro. We also report the production of soluble host factors including the upregulation of heparanase (HPSE) and inflammatory host factors such as interleukin-6 (IL-6), monocyte chemoattractant protein 1 (MCP-1), RANTES (regulated on activation, normal T cell expressed and secreted), interferon gamma (IFN-γ), and tumor necrosis factor alpha (TNF-α), which are also present during clinical disease in humans. Our study is the first to demonstrate that human chondrocyte cells are permissive to RRV infection, support the production of infectious virus, and produce soluble factors including HPSE, which may contribute to joint degradation and the pathogenesis of disease.

## 1. Introduction

The occurrence of rheumatic manifestations in viral diseases, mimicking those seen in degenerative osteoarthritis (OA) or autoimmune rheumatic arthritis (RA), is not an unfamiliar concept [1,2]. Prominently, arthritogenic alphaviral diseases (such as Ross River virus; RRV and chikungunya virus; CHIKV) have been described to have comparable clinical symptoms as well as both joint damage and inflammatory factor profiles as with those found in both OA and RA. For example, as reported in studies on the pathogenesis of OA, the expression of certain pro-inflammatory cytokines (interleukin-6; IL-6 and interleukin 1 beta; IL-1β) determines the degree of cartilage degeneration. These soluble mediators are also heavily upregulated in arthritogenic alphaviral disease, suggesting similar disease pathophysiology [3,4].

Human arthritogenic alphavirus infection results in a range of clinical manifestations, both acute and chronic phases of disease. Patients have debilitating movement impairment that ranges from symmetrical joint swelling of the peripheral joints in addition to limb arthralgia and myalgia [5]. Increasingly, multiple murine studies have also identified the pathological effects of arthritogenic alphavirus infections on the skeletal system, including thinning of cartilage and bone [6,7]. Recently, our laboratory reported substantial cartilage erosion with elevation of cartilage degrading matrix enzymes, such as a disintegrin and metalloproteinase with thrombospondin motifs 5 (ADAMTS5) in the joints of RRV-infected mice during peak disease. This was the first report showing that arthritogenic RRV infection influences cartilage breakdown in an ongoing infection [8]; however, the cellular source of these factors is largely unknown.

To further examine the relationship between alphaviral infections and cartilage breakdown, we analysed the joints of RRV-infected mice to identify which cell types are permissive to RRV infection. To relate this to the human condition, we further evaluated the permissibility of primary human chondrocyte and skeletal muscle cells to RRV and assessed the host cell responses to infection in these cell types.

## 2. Materials and Methods

### 2.1. Virus

Stocks of the RRV T48 strain were generated using an infectious clone containing the full-length sequence of T48 (a kind gift from Richard Kuhn, Purdue University), as described elsewhere [9]. Briefly, the plasmid pRR64 was linearised by SacI restriction enzyme digestion, followed by in vitro transcription using SP6 RNA polymerase and electroporation of the RNA into Vero cells for infectious virus production. Virus titers were quantified by plaque assay using Vero cells.

### 2.2. Mice and Histology

C57BL/6 wild-type mice obtained from the Animal Resources Centre (ARC) were infected subcutaneously in the thorax with 10^4^ plaque-forming units (PFU) of RRV or mock-infected with phosphate buffered saline (PBS). All animal experiments were conducted in strict accordance with the Griffith University Animal Ethics guidelines defined by Animal Ethics Committee (GLY/04/15; started 22 August 2015). Mice were monitored daily, weighed, and clinically scored for disease signs as described previously [8]. At Day 5 post-infection, mice were sacrificed, ankle joints were collected and decalcified in 14% ethylenediaminetetraacetic acid (EDTA) prepared with diethylpyrocarbonate (DEPC)-treated MilliQ water over a period of 7 days and fixed for 3 days in 4% paraformaldehyde (PFA).

Paraffin-embedded sections were cut (5 μm thick), deparaffinised using xylene and rehydrated through a series of alcohol washes (100%, 90%, and 70% ethanol). The tissue sections were permeabilised with Proteinase K for 20 min at room temperature, followed by incubation in prehybridisation buffer containing saline sodium citrate (SSC) and deionised formamide for 30 min at 37 °C. Subsequently, the tissue sections were incubated with hybridisation buffer containing a digoxygenin (DIG)-labelled riboprobe complementary to the conserved region of RRV structural envelope 2 protein (E2) for 24 h in a humidified chamber at 37 °C. After several washes with TBS-T (Tris-buffered saline with Tween 20), the slides were blocked using anti-mouse Fab antibody for 30 min and washed with TBS-T. Slides were treated with 2% hydrogen peroxide to block endogenous peroxidase activity. After a blocking step, the slides were incubated with anti-DIG-horseradish peroxidase (HRP) antibody for 30 min and washed with TBS-T. Tyramide signal amplification (TSA^®^) was performed to enhance the signal using tyramide reagents conjugated to fluorophores. Tyramide-FITC was used to amplify the signal from the anti-DIG-HRP antibody.

The slides were washed with TBS-T to remove unbound tyramide reagent, blocked, probed for type II collagen using rabbit anti-type II collagen antibody, and detected using anti-rabbit-HRP antibody with tyramide-Cy3. This was repeated in a similar manner for the detection of β-tubulin using tyramide-Cy5. The tissue sections were stained using 4′,6-diamidino-2-phenylindole (DAPI), washed with TBS-T, and mounted using fluorescence mounting media. To rule out non-specific RNA probe binding, a negative control probe of similar size was designed using the sequence of dengue virus serotype 2 strain (DENV-2/SG/D2Y98P-PP1/2009; Genbank JF327392.1) to demonstrate the specificity of the RRV E2 probe.

Images were captured using a DeltaVision/Olympus IX70 microscope system (Applied Precision, Issaquah, WA, USA) and analysed using ImageJ software (version 1.51s, National Institutes of Health, Bethesda, MD, USA). The intensity of the fluorescein isothiocyanate (FITC) signal was quantified by measuring the integrated density of the chondrocyte cells in the tissue section. Regions of equal size were used for the quantification, and 5 regions each were measured in 2 different image views per sample.

### 2.3. Primary Cell Cultures and Infection

Primary human chondrocyte and skeletal muscle cells (Clonetics, Lonza, Walkersville, MD, USA) were cultured in 12-well plates. Chondrocytes were differentiated according to the manufacturer’s instructions, and cells were then infected with RRV at a multiplicity of infection (MOI) of 1.0 for 1 h at 37 °C. The virus innoculum was removed, and cell monolayers were washed once with PBS. After the addition of 1 mL of maintenance media to each well, the cells were further incubated at 37 °C. Cell culture supernatants were collected at time-points 0, 6, 12, 24, 36, and 48 h post-infection (h.p.i.), clarified by benchtop centrifugation to remove cellular debris and used for the determination of virus titer via plaque assay. Total RNA was extracted from the cells at each time-point using TRIzol reagent (Life Technologies, Waltham, MA, USA) according to manufacturer’s instructions and used for gene expression analysis by real-time PCR. Samples collected from triplicate wells were used for the analysis. Primary human chondrocyte cells used for all experiments were re-differentiated for 7 days prior to virus infection. The re-differentiated chondrocyte cells were cultured on cover slips, washed 3× with PBS, and fixed using 4% PFA in PBS. After washing 3× with PBS and blocking with 1% bovine serum albumin (BSA) in PBS, the cells were characterised for type II collagen expression using rabbit anti-type II collagen antibody. The cells were subsequently washed 3× using PBS and incubated with anti-rabbit-HRP antibody. Positive signal was detected using 3,3′-diaminobenzidine (DAB) substrate.

### 2.4. Gene Expression Analysis by qPCR

The isolated total RNA (1 µg) was reverse-transcribed to produce cDNA using iScript Reverse Transcription Supermix kit (Bio-Rad, Hercules, CA, USA). Commercially available QuantiTect primers (Qiagen, Hilden, Germany) for IL-6, monocyte chemoattractant protein-1 (MCP-1), and IL-1β were used and primer sequences for a disintegrin and metalloproteinase with thrombospondin motifs 4 (ADAMTS4), a disintegrin and metalloproteinase with thrombospondin motifs 5 (ADAMTS5), aggrecan (ACAN), type I collagen (COL1A1), type II collagen (COL2A1), heparanase (HPSE), matrix metalloproteinase-3 (MMP3), matrix metalloproteinase-9 (MMP9), and tissue inhibitor of metalloproteinase 3 (TIMP3) genes obtained from existing literature (Table 1). Real-time PCR was performed using SYBR Green supermix (Bio-Rad). Relative gene expression was expressed as fold change in gene expression between mock-infected and RRV-infected samples, with threshold cycle (C_T_) values normalised using the HPRT1 housekeeping gene (QuantiTect primer, Qiagen).

### 2.5. Bio-Plex^®^ Multiplex Assay

Levels of cytokines were analysed using the Bio-Plex^®^ Multiplex Immunoassay system (Bio-Rad, Hercules, CA, USA). Data acquisition was done using a Luminex 200 (Bio-Rad) and analysed using the Bio-Plex^®^ Manager 6.1 software (Bio-Rad).

### 2.6. Statistical Analysis

Viral titers are represented as means of triplicate samples with standard error of mean (SEM). Gene and protein expression data for chondrocytes and skeletal muscle cells are represented as means of triplicate samples with SEM and analysed by two-way ANOVA statistical analysis with Bonferroni’s test. Gene expression levels and FITC intensity in chondrocytes were analysed by one-way ANOVA followed by Dunnett’s multiple comparisons test.

## 3. Results

### 3.1. Murine Chondrocytes Are Susceptible to RRV Infection

**Figure 1:**
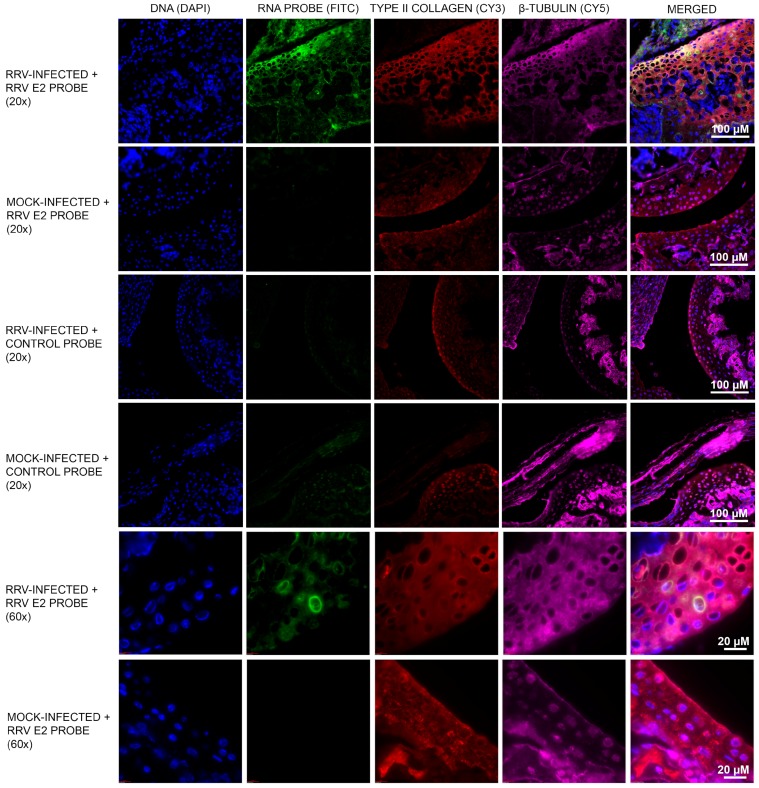
Murine chondrocyte cells are susceptible to Ross River virus (RRV) infection. Chondrocyte cells in the cartilage region of RRV-infected mice were stained positive for RRV envelope 2 (E2) RNA by fluorescence in situ hybridisation (green/FITC). To identify the chondrocyte cells, type II collagen immunostaining was performed to locate the cartilage region (red/Cy3). Tissue sections were also stained to better visualise cell morphology: DNA (blue/DAPI) and β-tubulin (magenta/Cy5). No FITC signal was observed in mock-infected mice. The experiment was also performed using a negative control probe to rule out any non-specific RNA probe binding. Images were captured at 20× and 60× magnifications.

Mice were infected subcutaneously with 10^4^ PFU RRV or mock PBS controls. At Day 5 post-infection, mice were sacrificed, and ankle joints were decalcified in 14% EDTA in DEPC-treated MilliQ water over a period of 7 days and fixed in 4% PFA (RNase-free). Paraffin-embedded sections (5 µm thick) were cut and probed with a DIG-labelled riboprobe complementary to a conserved region of RRV E2. RRV-positive chondrocytes were observed within the ankle joints of infected mice (green/FITC; Figure 1). No FITC signal was observed in mock-infected mice and for the negative control probe tested. As chondrocyte cells are the main cell type in cartilage tissue, type II collagen, a cartilage-specific marker, was used to identify the cells in this region as chondrocytes (red/Cy3; Figure 1). The fluorescence intensity (IntDen) was quantified using ImageJ software and the FITC signal from the RRV E2 probe in RRV-infected sample was deemed statistically significant (*p* < 0.0001) (Figure S1).

### 3.2. Primary Human Chondrocyte and Skeletal Muscle Cells Are Permissive to RRV Infection and Supports Productive Virus Replication

**Figure 2:**
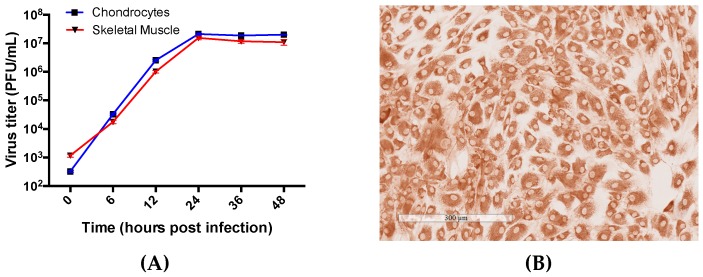
Human chondrocyte cells are susceptible to Ross River virus (RRV) infection. (**A**) In vitro virus titers (PFU/mL) of RRV-infected human chondrocyte cells (blue) and human skeletal muscle (red) cells at time-points 0, 6, 12, 36, and 48 h post-infection (h.p.i.) with data represented as means of triplicate samples with SEM. (**B**) Characterisation of type II collagen expression in human chondrocyte cells (brown). Image taken at 10× magnification.

To determine if RRV replicates in chondrocytes and skeletal muscle, human primary cells were cultured and infected at an MOI of 1. Cell culture supernatants were collected at time-points over a 48 h period and viral titers were determined by plaque assay. The cells produced increasing amounts of infectious virus particles with viral titers peaking at 24 h.p.i. in both cell types (Figure 2A). This illustrates that RRV is able to enter the cells, replicate its genome and structural proteins required for virus assembly, and bud out of the cells successfully. Type II collagen expression was characterised in human primary chondrocyte cells as shown in Figure 2B.

### 3.3. Upregulation of the Genes Encoding Pro-Inflammatory Factors and Degrading Enzymes Was Observed

**Figure 3:**
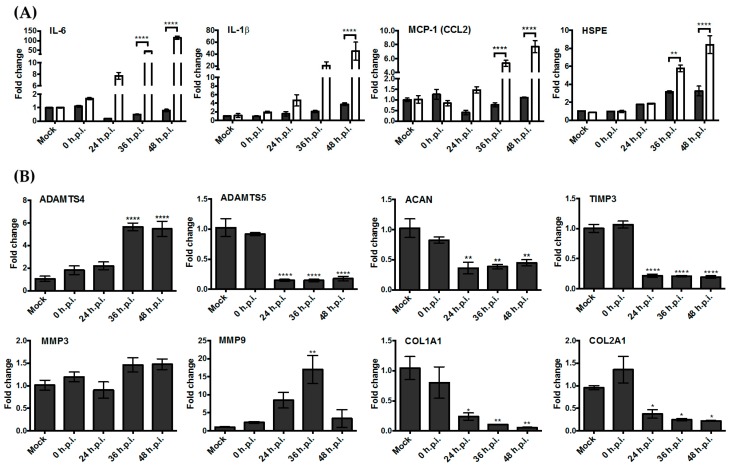
Upregulation of key pro-inflammatory genes and extracellular matrix breakdown genes during Ross River virus (RRV) infection in human chondrocyte cells (grey) and skeletal muscle cells (white) at 0, 24, 36, and 48 h post-infection (h.p.i.). Samples were normalised using values from mock-infected groups. (**A**) Relative gene expression of key pro-inflammatory markers. Statistical analysis was performed by two-way ANOVA statistical analysis with Bonferroni’s test. (**B**) Relative gene expression of articular cartilage components and associated breakdown markers. Statistical analysis was performed by one-way ANOVA followed by Dunnett’s multiple comparisons test. * *p* < 0.05, ** *p* < 0.01, **** *p* < 0.0001.

To assess the impact of RRV infection on gene expression in human chondrocytes and skeletal muscle cells, their gene expression profiles of pro-inflammatory cytokines and extracellular matrix degrading enzymes were studied by qPCR. Among the genes surveyed, we observed upregulation of IL-6, MCP-1, and IL-1β over time in skeletal muscle (Figure 3A) cells. We also noted elevated gene expression levels of heparanase in both skeletal muscle cells and chondrocyte cells (Figure 3A). Heparanase is an enzyme that breaks down heparan sulfate and is associated with both joint pathologies and the regulation of cytokine signalling [14].

To determine if RRV contributes to damage of the extracellular matrix (ECM), we assessed the gene expression of several ECM components found in the articular cartilage and their associated breakdown markers (Figure 3B). Following RRV infection in human chondrocyte cells, aggrecan, type I collagen, and type II collagen, which are ECM components, were observed to be downregulated. Among the enzymes known to cause ECM breakdown, ADAMTS4 and MMP9 were found to be upregulated.

### 3.4. Key Pro-Inflammatory Soluble Mediators Such as IL-6, MCP-1, RANTES, IFN-γ, and TNF-α Were Produced during RRV Infection

**Figure 4:**
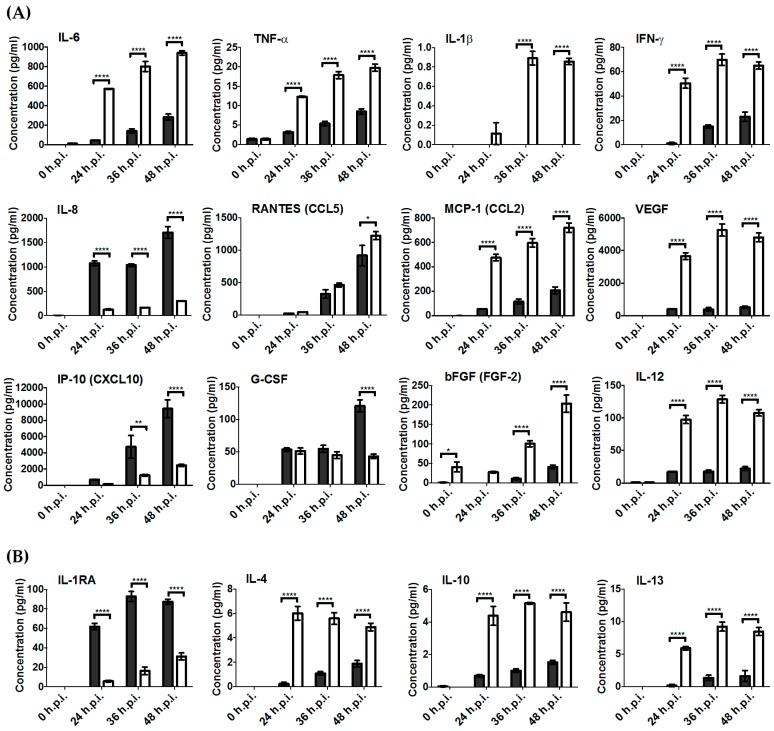
Soluble mediators present during Ross River virus (RRV) infection in human chondrocytes cells (grey) and skeletal muscle cells (white) primary cells at 0, 24, 36, and 48 h post-infection (h.p.i.). (**A**) Pro-inflammatory and (**B**) anti-inflammatory cytokine levels were measured using the Biorad Bio-Plex^®^ multiplex system. Statistical analysis was performed by two-way ANOVA statistical analysis with Bonferroni’s test. * *p* < 0.05; ** *p* < 0.01, **** *p* < 0.0001.

To further confirm the level of inflammatory factors produced in RRV-infected chondrocyte and skeletal muscle cells, we assessed the concentration of soluble cytokines produced using the Bio-Plex^®^ Multiplex Immunoassay system (Bio-Rad). We found elevated protein expression of many pro-inflammatory cytokines associated with RRV infection such as IL-6, MCP-1, RANTES, IFN-γ, and TNF-α (Figure 4A). Similar to results obtained via gene expression studies, a more significant change in the levels of cytokines was observed in skeletal muscle cells compared to chondrocytes. We also found upregulation of anti-inflammatory cytokines such as IL-1RA, IL-4, IL-10, and IL-13, suggesting that there may be mechanisms in place for cytokine regulation during RRV infection (Figure 4B).

## 4. Discussion

Chondrocytes are the main cell type found in cartilage tissue and are essential for maintaining the cartilaginous matrix. They are involved in not only the pathogenesis of non-inflammatory arthritis, such as osteoarthritis, a degenerative articular cartilage disease, but also inflammatory arthritis, such as rheumatoid arthritis, an autoimmune disease [18]. Interestingly, there are sporadic reports showing that chondrocytes are also susceptible to virus infections and play an important role in the related disease pathogenesis. Human cytomegalovirus (AD169 strain) was able to infect chondrocytes, which led to extensive cytopathic effects including cell aggregation, fusion, and lysis, suggesting that the related etiopathogenesis of articular diseases may involve chondrocytes [2]. Similarly, Rous sarcoma virus (RSV), an oncovirus that causes sarcoma in avian species, was shown to be able to infect chondrocytes, and this resulted in reduced type X collagen synthesis, proteoglycan, and calcification of the extracellular matrix, suggesting that RSV infection was able to disrupt chondrocytes differentiation and mineralisation [19]. Human endogenous retrovirus transcripts were also found in chondrocytes and cartilage from osteoarthritis patients, but their role in disease pathogenesis is currently unknown [20].

In the field of gene therapy, chondrocytes were reported to be permissive to adeno-associated virus, based on which a gene delivery therapy was established for articular joint disorders [21]. Though there are no direct reports on the role of chondrocytes in arthritogenic alphaviral-induced articular cartilage diseases, a study on CHIKV-infected interferon response factors 3 and 7 knockout (IRF3/7^−/−^) mice demonstrated evidence of viral RNA present in articular cartilage, which was not identified in CHIKV-infected wild-type mice [22]. Additionally, earlier studies on Ross River virus disease (RRVD) found the presence of RRV in murine joint tissues such as the tendons, ligaments, and synovial tissue, but these studies do not identify chondrocytes or cartilage as a source of virus [23,24]. Many studies have also reported that RRV-induced inflammatory disease comprises both myositis in skeletal muscle and involvement of osteoclastogenesis and bone reabsorption [6,23,25,26,27]. Therefore, we speculate that RRV infection in chondrocytes could play a role in disease pathogenesis. Here, we show for the first time that (i) RRV is present in chondrocytes of infected wild-type mice in vivo, (ii) RRV is able to replicate robustly in primary human chondrocytes, and, more importantly, (iii) these infected primary cells secreted significant amounts of inflammatory soluble factors. Pro-inflammatory cytokines such as IL-1β, TNF-α, MCP-1, and IL-6 have been previously shown to contribute to the RRV-induced inflammation, but this is the first report indicating that chondrocytes may be a source of these factors. Furthermore, the upregulation of heparanase following RRV infection is a novel discovery [1,2,3]. Therefore, infected chondrocytes are likely to play important roles in RRV disease pathogenesis by both amplifying infection and through the production of soluble factors. Further studies are needed to elucidate the mechanisms of immunological processes and the potential role of chondrocytes and the articular cartilage in alphaviral-induced arthritis.

## Figures and Tables

**Table 1:** Primer sequences used in gene expression studies.

Gene	Primer Sequence	Reference
ADAMTS4	F: 5′-AGG CAC TGG GCT ACT ACT AT-3′ R: 5′-GGG ATA GTG ACC ACA TTG TT-3′	[10]
ADAMTS5	F: 5′-TAT GAC AAG TGC GGA GTA TG-3′ R: 5′-TTC AGG GCT AAA TAG GCA GT-3′	[10]
ACAN	F: 5′-TCG AGG ACA GCG AGG CC-3′ R: 5′-TCG AGG GTG TAG CGT GTA GAG A-3′	[11]
COL1A1	F: 5′-AGG TGC TGA TGG CTC TCC T-3′ R: 5′-GGA CCA CTT TCA CCC TTG T-3′	[12]
COL2A1	F: 5′-ATG AGG GCG CGG TAG AGA C-3′ R: 5′-CGG CTT CCA CAC ATC CTT AT-3′	[13]
HPSE	F: 5′-TGG ACC TGG ACT TCT TCA CC-3′ R: 5′-TTG ATT CCT TCT TGG GAT CG-3′	[14]
MMP3	F: 5′-GAC AAA GGA TAC AAC AGG GAC CAA T-3′ R: 5′-TGA GTG AGT GAT AGA GTG GGT ACA T-3′	[15]
MMP9	F: 5′-GCC ATT CAC GTC GTC CTT AT-3′ R: 5′-TTG ACA GCG ACA AGA AGT GG-3′	[16]
TIMP3	F: 5′-ACG ATG GCA AGA TGT ACA CAG G-3′ R: 5′-GGA AGT AAC AAA GCA AGG CAG G-3′	[17]

ADAMTS: a disintegrin and metalloproteinase with thrombospondin motifs; ACAN: aggrecan; COL1A1: type I; COL2A1: type II collagen; HPSE: heparanase; MMP: matrix metalloproteinase; TIMP3: tissue inhibitor of metalloproteinase 3.

## Supplementary Materials

Supplementary materials can be found at http://www.mdpi.com/1999-4915/10/2/86/s1.
