# Supplementary material for: Chondrocytes Contribute to Alphaviral Disease Pathogenesis as a Source of Virus Replication and Soluble Factor Production
Source: Viruses. 2018 Feb 15;10(2):86. doi: 10.3390/v10020086 (PMC5850393; doi:10.3390/v10020086)
Supplement: Supplementary file 1 [file viruses-10-00086-s001.pdf]

Supplementary material

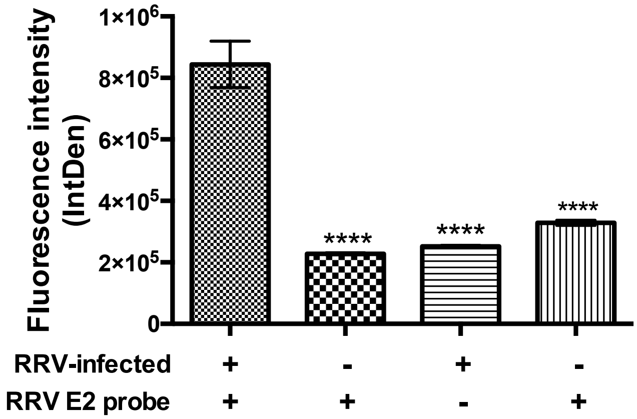

Figure S1. Murine chondrocyte cells are susceptible to RRV-infection. Fluorescence intensity of DIG-labelled RRV-specific RNA probe to joint sections of RRV-infected and mock-infected (PBS) mice. A negative control probe was also used to evaluate any non-specific RNA probe binding. Integrated density (IntDen) values were expressed as means with SEM. Statistical analysis was performed by one-way ANOVA followed by Dunnett's multiple comparisons test. *p<0.05; **p<0.01, ***p<0.001, ****p<0.0001.
